# Fighting isn’t sexy in lekking greater sage-grouse: a relational event model approach for mating interactions

**DOI:** 10.1098/rspb.2024.2981

**Published:** 2025-05-21

**Authors:** Samuel S. Snow, Gail L. Patricelli, Carter T. Butts, Alan H. Krakauer, Anna C. Perry, Ryane Logsdon, Richard O. Prum

**Affiliations:** ^1^ Institute for Advanced Study in Toulouse, University of Toulouse 1 Capitole, Toulouse, Occitanie, France; ^2^ Ecology & Evolutionary Biology, Yale University, New Haven, CT, USA; ^3^ Department of Evolution and Ecology and Animal Behavior Graduate Group, University of California Davis, Davis, CA, USA; ^4^ School of Social Sciences, University of California Irvine, Irvine, CA, USA

**Keywords:** relational event model, lek, social network, male aggression, mate choice

## Abstract

The relationship between aggression and mate choice in mating systems is critical for understanding the evolution and diversification of sexual organisms and yet remains the subject of vigorous debate. A key challenge is that traditional correlational approaches cannot distinguish underlying mechanisms of social interaction and can indicate misleading positive associations between aggression and mating events. We implement a novel relational event model (REM) incorporating temporal dependencies of events in a social network to study natural reproductive behaviour in a lek-breeding system where males gather to display and females visit to evaluate mates, often observing both male courtship displays and fights. We find that fighting is not attractive to females. Indeed, males are less likely to start and more likely to leave fights with females present, plausibly to avoid entanglement in protracted combat cycles arising from emergent social processes that reduce availability to mate. However, fighting serves other roles, e.g. to deter copulation interruptions and rebuff competitors. Our findings support the hypothesis that social systems regulating conflict and promoting females’ choice based on display are fundamental to stable lek evolution. Moreover, our analysis highlights the utility of the REM framework in testing mechanistic hypotheses in behavioural ecology and evolution.

## 1. Introduction

Determining whether and when aggression is aligned or in conflict with the free choice of mates is crucial for understanding underlying evolutionary pressures and processes in mating systems, including the source and strength of selection on sexual ornaments and behaviours [1–6], our interpretation of diversity in sex roles [7] and expectations for the production of biodiversity and maintenance of species boundaries [8–10]. Pinpointing causal mechanisms by which (usually male) competition and aggression influence male mating success and the extent to which they constrain or promote female mate choice is especially important in light of recent theoretical work showing how male aggression that hinders the efficacy of female mate choice can produce selection for female behaviours or morphologies that resist those effects—even when a positive association exists between attractiveness and aggression (i.e. when attractive males are also more aggressive) [11]. Selection for resistance behaviours can lead to very different expected outcomes in the evolutionary trajectory of mating interactions, as compared to models that assume male aggression and female mate choice are aligned [11–13].

Teasing apart various ideas about the role of aggression in mating systems (and understanding social behaviours in general) is notoriously difficult, and much of the challenge is analytical. Mating success and histories of aggressive behaviour are the result of episodic social interactions that unfold over time. Yet in evolutionary biology, we typically aggregate these social events into counts, or, increasingly, metrics for behaviours embedded in a social network [14] and then correlate those metrics with outcomes like mating success [15–18]. There has been a growing recognition that our ability to understand the processes underlying complex social dynamics within groups of individuals hinges on our understanding of how social network structures and relationships change over time [19–22], including within animal social [23–26] and reproductive [27,28] systems explicitly involving the interplay of aggression, cooperation and mate choice. However, previous dynamic approaches (such as time-ordered networks [23,29], temporal exponential random graph models [22,30] and stochastic actor-oriented models [19,20,26,27]) all rely on researchers choosing a temporal window over which to aggregate social events. This temporal binning leads to a loss of information on the timing and sequence of events [31,32], making it difficult to ask questions about mechanistic relationships among behaviours. For example, eliminating information on the sequence and timing of fighting and mating events would cause us to implicitly (and inappropriately) assume that many females are making mating decisions using information about fights that have not yet occurred and to recover potentially misleading correlations between fighting and mating success with respect to underlying mechanisms.

**Figure 1.**
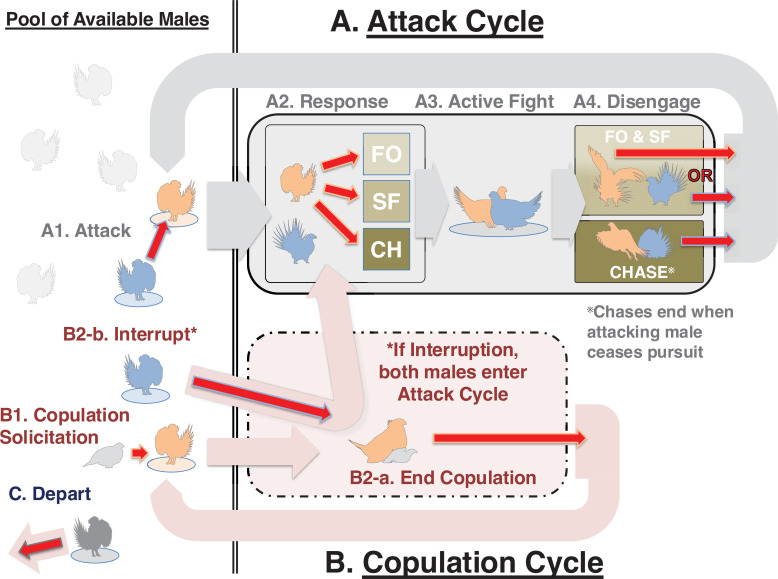
Event scheme and model constraints for the REM. Red arrows are endogenous event categories for which hazards are estimated. Wide arrows represent transitions of males into, through and out of behavioural cycles. When an attack occurs (A1), the attacking and receiving males enter the attack cycle (A; solid box) and remain unavailable for other interactions until a disengagement, at which point they return to the pool of available males (A4; wide grey arrow). The receiver’s immediate response (A2) determines whether the interaction is a face-off (FO), short fight (SF) or chase (CH). Disengagement (A4) outcomes depend on the interaction type: either male can disengage from a FO or SF, but only the attacker may disengage from a chase, winning it. When a female solicits copulation (B1), the solicited male enters a copulation cycle (B; dashed box). Copulating males remain unavailable until the copulation ends, and they return to the pool (B2-a). However, another male may interrupt the copulation by attacking the copulating male (B2-b), breaking the copulation cycle and initiating an attack cycle. Any available male can depart the lek (C).

To address this problem, we have for the first time implemented a relational event model (REM) to study natural reproductive behaviour [31–36]. REMs comprise a statistical framework that explicitly models the temporal dependencies within sequences of social events among a network of individuals unfolding in continuous time. For every point in time, we model a *hazard*, or conditional rate of occurrence, for every behavioural event that is possible at that moment, such as ‘male A attacks male B’ or a solicitation for copulation (figure 1 and electronic supplementary material, figure S1). The event hazards are a function of the past observed event history, a set of predictor statistics encoding various mechanisms that may affect the hazards and a set of parameters that govern the magnitude and direction of the effects (equation (2.1)). We estimate the most probable parameter values for each predictor given the observed data, ultimately choosing the best model from among models built with different possible predictor sets. In this way, the REM framework enables us to directly investigate potential mechanistic relationships among events across multiple timescales and combinations of behaviours.

We used detailed observations of a wild population of greater sage-grouse (Aves: *Centrocercus urophasianus*), an iconic lek-breeding species where males gather to display, and females visit to evaluate and choose mates. The relationship between male competition and female mate choice in the context of lek-breeding has long been the subject of vigorous debate. Numerous evolutionary scenarios have been proposed to explain the evolution of lekking and its associated spatial clustering of courters, extreme mating skew and often spectacular courtship displays, many hinging fundamentally on the extent and direction of the influence of male competition on female mate choice [6,15,37–46]. Competitive interactions among males on leks are often directly observable by females alongside courtship displays, yet in many lekking species, including greater sage-grouse, males often also fight when females are absent (affording no possibility of direct evaluation by females), and will frequently attack during mating attempts, possibly endangering females and in seeming conflict with females’ choices [40,47–52].

To take a new look at the role of male–male aggression in greater sage-grouse, we built REMs to investigate how particular histories, patterns or outcomes of fights among males and various other factors such as female presence, predict involvement in future fights, fight outcomes, solicitations for copulations (coupled with subsequent mounting by the male; see §2d) and copulation interruptions. Previous studies of lekking birds have found positive statistical associations between aggression and mating success, leading some to suggest that fighting itself is an attractive display indicative of mate quality and that aggregations on leks provide opportunities for females to view or even incite contests for adaptive benefit [1,15,18,37,38,53,54]. To investigate the idea of fighting as an attractive or informative element of male display, we fit the following two different base models: (i) the ‘Aggression’ model, which employs a single fixed effect for the solicitation hazard for all males, essentially forcing the REM to use aspects of fighting events to directly explain variation in propensity of copulation events for each male, and (ii) the ‘Differential Attractiveness’ model, which uses individual-level fixed effects for solicitation hazards, allowing the model to attribute variation in solicitation across males to some intrinsic property, such as their display attractiveness, in addition to (and independent of) the possible effects of their fighting behaviour.

Aggression could be unrelated or negatively related to female solicitations directly, yet still be potentially positively associated with individual male mating success via diverse indirect mechanisms. Using the REM, we examine the temporal interdependencies of different fighting behaviours such as fights without females present and copulation interruptions, potentially revealing a social regulatory role for aggression in certain contexts and/or self-regulation of aggression that could indirectly contribute to mating success. Significantly, because the empirically calibrated REM leads directly to a generative model, it also allows us to probe the consequences of interaction mechanisms for the overall social system through simulation. In particular, we can examine the longer-term consequences of fighting for both copulation opportunities and future combat via indirect, emergent mechanisms, such as persistent cycles of conflict that, once initiated, are difficult to escape.

We show that fighting serves a complex and context-dependent role in this system. Our results suggest that the avoidance of fights and the overall regulation of aggression is likely necessary for a stable, functioning lek, with important implications for our understanding of lek evolution.

## 2. Methods

### (a) Field data collection

We conducted daily observations from 11 March to 4 May 2014 at the ‘Chugwater’ lek, southeast of Hudson, Wyoming, USA. We video-recorded on-lek activity with one or two HD cameras (1080i; Sony HDR-HC1 and Canon Vixia HV40) from first light until all birds departed. Field assistants recorded videos from a blind approximately 75 m away and documented individual males’ positions on the lek relative to a grid of stakes arrayed at 10 m intervals, covering the centre of activity [55,56]. Males were identified via unique colour band combinations and tail plumage patterns [47,57].

### (b) Video data extraction

We extracted behavioural data from videos recorded daily from 19 March to 6 April 2014, excluding 27 March due to poor weather, giving 18 video days of data. This period coincided with peak breeding activity, capturing the entire first-clutch breeding pulse with 72 of 85 total copulations observed over the whole season. Often there is a second, subsequent breeding pulse following some broods’ failure when those females return to re-mate [47] (G.L.P., personal observation). The first pulse thus likely represents females’ first choices.

Research assistants were trained and vetted for precision using a standardized protocol (available in the electronic supplementary material). We described details and timing (to the nearest frame, 1/30 s) of every interaction between males and all copulations with females. Nearly all male interactions were agonistic; these ‘fights’ are defined and described in electronic supplementary material, figure S1.

We also recorded each male’s arrival on the lek, as well as the females’ entrance (first female to appear) and exit (last female’s departure). We defined ‘female presence’ as when any females are on the lek. Disturbances causing birds to flush prematurely (e.g. predators and aeroplanes) were documented, totalling four events. Males’ positions relative to the stake array were noted when they either initiated or received an attack and at 5 min intervals independent of their activity to estimate territories [58] and movement centroids.

We recognized the following three categories of males: identified (positively identified and having persistent IDs throughout the season), unknown ‘U males’ (consistently present on the lek, but only for a single day or unidentified on that day) and transient ‘X males’, usually juveniles/yearlings [47]. Females, though distinguishable from males by plumage, could not be individually identified.

Typically, several sage-grouse males establish specific lek territories, faithfully returning each day [47]. For each video day, we defined territory-holding males as those present for at least the previous two consecutive days.

The dataset ultimately included 1506 unique interactions among up to 29 individual males (14 identified, 15 ‘U’), plus ‘X’ males. On average, 8.56 (±2.01) males were present daily, and females attended for at least one interval every day. We observed 72 copulation events, 22 disrupted by other males, an interruption rate typical of greater sage-grouse [47,49].

### (c) Relational event model

The REM directly models the unfolding of event sequences (e.g. observed social behaviours) over time to identify the underlying processes that may have generated them. It estimates a hazard for each possible event at any given moment. Hazards, representing an event’s propensity to occur, are conditional rates: impossible events have a hazard of 0, while more frequent events have higher hazards. Event hazards may depend on past events, event type and/or covariates. To capture this, hazards are modelled as multiplicative functions of predictor statistics, which encode the effects of mechanisms such as sender/receiver identity or the influence of historical events (e.g. recent attacks) that might enhance or inhibit the rate of occurrence [32].

We formalize the expression for an event hazard as follows (adapting from [32]): given a set of event types *C*, we can define a single event *a* as a list of elements containing the type of event *c* = *c*(*a*) ∈ *C* and the time the event occurred *τ* = *τ*(*a*), such that *a* = (*c*, *τ*). The history of past events from time 0 leading up to time *t* is denoted as *Α_t_* = {*a_i_*: *τ*(*a_i_*) ≤ *t*}, and A is the set of possible events at any moment. *X_a_* is a set of covariates that may be associated with a given event *a*, such as the categories of the males (identified, ‘U’ or ‘X’) and the individuals’ spatial proximity. We can then express the hazard, *λ*, of an event *a* at time *t*, given event history *Α_t_* as


(2.1) $$\lambda_{aA_{t}\theta }=\left\{ \begin{array}{l} \exp\left(\theta^{T}u\left(c\left(a\right),X_{a},A_{t}\right)\right)\;\;\;\;\;\;if\;a\in A\left(A_{t}\right)\\ \;\\ 0\;\;\;\;\;\;\;\;\;\;\;\;\;\;\;\;\;\;\;\;\;\;\;\;\;\;\;\;\;\;\;\;\;\;\;\;\;\;\;\;\;otherwise, \end{array}\right.$$

where θ is a vector of parameters governing the effects of u, a vector of predictor statistics representing the effects of mechanisms that may influence the hazard of a given event. The set of predictor statistics (u) may be functions of the event type and/or past events. Each unit change in $u_{i}$ multiplies the hazard of the event by $exp(\theta_{i})$. In this way, the predictor statistics comprising u are analogous to the effects in a conventional hazard model [59], with θ being the model coefficients inferred from data.

As is typical with REMs, we assume the system’s state—the set of possible events and their associated hazards—is ‘piecewise constant’, with changes only precipitated by new events. For example, a new event, such as an attack, might affect the propensity of other actions and/or limit the individuals available for interaction going forward. We update the system’s state to reflect this, and then the context is updated again when a new event occurs, such as a disengagement from the fight. This is reflected in equation (2.1): the hazard does not directly depend on *t* but rather on the temporally ordered event history. Piecewise constancy is tantamount to the assumption that the waiting time between events is conditionally exponentially distributed, meaning that an event with hazard *λ* at time *t*, given a prior event at time *t*′, has a survival function $S\left(t\right)=e^{-\lambda \left(t-t^{\prime }\right)}$, and, accordingly, holding other factors constant, the expected waiting time is 1/*λ*. The probability of a particular event being the one to occur next (and at a particular time) is dictated by its hazard in proportion to the total hazard for all possible events. Exogenous events [33] add flexibility within the piecewise-constant model assumption, allowing for events to occur that are not endogenous but nevertheless affect the system, or for bookkeeping devices such as ‘clock’ events, which allow event hazards to potentially depend on time directly. We incorporate both here (see §2d).

We test various hypotheses regarding aggression in sage-grouse by selecting among possible models (different sets of predictor statistics comprising alternative u vectors) and assessing goodness-of-fit to the observed event sequence (see expression for model likelihood under the above-described process, incorporating exogenous effects, in Marcum *et al*. [33]). We conducted all REM analyses using the ‘relevent’ package for R [60,61] using Bayesian estimation of posterior modes to estimate model coefficients. The final models reported below, respectively, identify subsets of the possible predictor statistics that, through their effects on event hazards governed by inferred parameter magnitudes, provide the most probable fit to the observed data as indicated by the Bayesian information criterion (BIC). Reproducible code is provided in the electronic supplementary material [62].

### (d) Event types

To effectively implement the REM, we operationalized and incorporated both observed behaviours and events that might influence them. Although most event types map onto dyadic sender/receiver ‘ties’ typical in dynamic social networks, our event set also includes unique categories.

**Figure 2.**
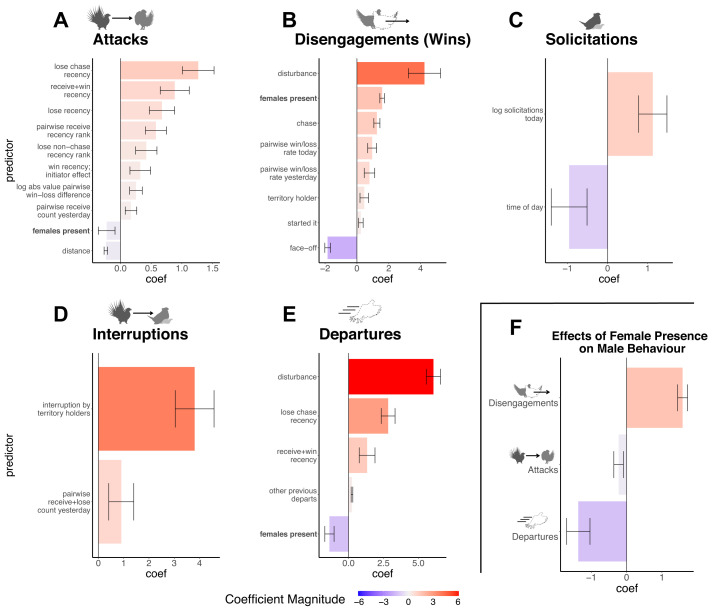
(A–E) Estimated coefficients for the predictor statistic set comprising the best-fitting ‘Differential Attractiveness’ model, arranged by event category affected. Positive (redder) coefficients increase the hazard (propensity of occurrence) of events when applicable; negative coefficients are the opposite. Attacks (A) and interruptions (D) refer to the hazard of a male receiving an attack or a copulation interruption, respectively, apart from statistics labelled ‘initiator effects’. Inset (F) highlights the effects of female presence, corresponding to bold-indicated predictors in (A,B,E). Error bars represent twice the Bayesian posterior estimated SEs, approximating 95% CIs. See electronic supplementary material, table S3, for full model results including fixed effects.

**Figure 3.**
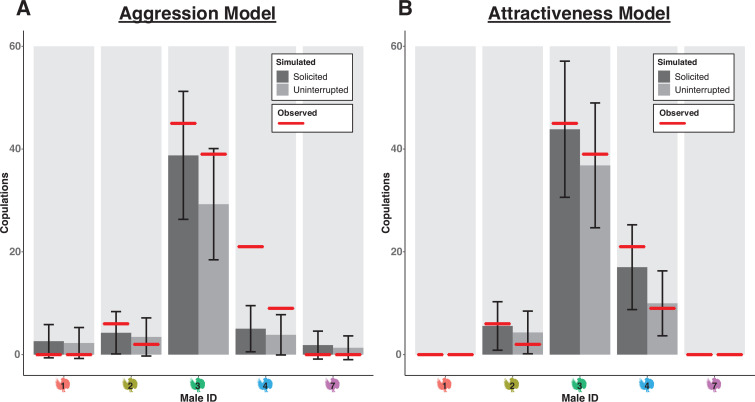
Simulated predictions of the numbers of solicitations and uninterrupted copulations generated from the alternative best-fitting ‘Aggression’ model (A) and ‘Differential Attractiveness’ model (B). Plots show mean total solicitations (dark bars) and mean total uninterrupted copulations (lighter bars) for each male across 100 simulations for each day of the observation period. Red lines are the observed empirical values. Error bars represent one s.d. The attractiveness model (B) yields a closer fit to the observed data. Data shown are for the five individuals that were present all days.

**Figure 4.**
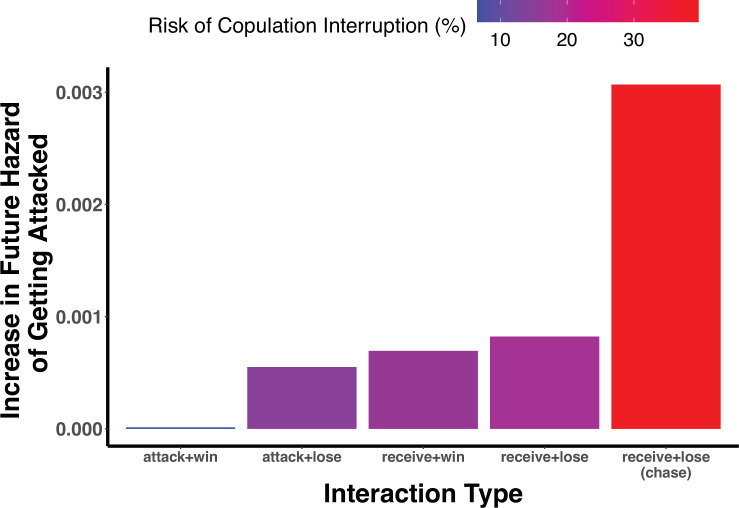
Effect of fight types on a male’s immediate hazard of getting attacked (*y*-axis) and the probability of interruption during copulation (colour scale). Values are based on coefficients from the best-fitting ‘Differential Attractiveness’ REM, simulating six territory-holding males with average attack rates, pairwise distances, no prior history and females present. Fights are combinations of initiation (attack or receive; electronic supplementary material, figure S1A) and disengagement (win or loss; electronic supplementary material, figure S1C). All fights are short fights (electronic supplementary material, figure S1B) except for the rightmost bar, which shows the increased attack risk after a chase (electronic supplementary material, figure S1B).

First, we defined an event type for each of the 870 pairwise permutations of the 29 males plus ‘X’ males attacking/receiving (electronic supplementary material, figure S1A, and figure 1A-1). To allow for fights of different kinds, we introduced ‘response’ events to immediately follow an attack, where the attacked male’s reaction defines the fight’s nature—run away and begin a ‘chase’, or stand their ground, eliciting a ‘short fight’ or a ‘face-off’ (electronic supplementary material, figure S1B, and figure 1A-2). Each fight concludes with a male-specific disengagement event (electronic supplementary material, figure S1C, and figure 1A-4). During a fight, often one male will attempt to disengage (turn away) but their interaction partner will lunge towards them, forcing them to continue. Accordingly, the ‘winner’ of a sage-grouse fight is considered the first male to successfully disengage [18,63] (electronic supplementary material, figure S1C). Thus, each ‘fight’ is an interval consisting of three ordered events: an initiating attack, an instantaneous response and sometime later, a disengagement (an ‘attack cycle’; figure 1A).

Copulations are bounded by the following two events (electronic supplementary material, figure S1D and figure 1B-1, B-2-a): (i) the start—a female’s solicitation followed by a male mounting—and (ii) an end copulation event (the male dismounting). Males are never observed to successfully copulate without a female’s solicitation. Occasionally a female may solicit, but the male does not mount immediately and continues courting; because female solicitations without copulations can be difficult to discern, here we only include events where a solicitation led to mounting. Thus, we defined ‘solicitation’ events as beginning at the moment the male mounts. Copulation interruptions (electronic supplementary material, figure S1E, and figure 1B-2-b) are defined as an attack on a copulating male prior to, and supplanting, a successful copulation end event.

We also included ‘exogenous’ event types—events with hazards independent of the behavioural events in the system. These include male arrivals (treated as exogenous, while departures, affected by events on the lek, are endogenous) and female entrances and exits, which dictate female presence. Additionally, we employed ‘clock’ events, scheduled to exogenously occur every 15 min. This lets the piecewise-constant system to update hazards based on time *per se*, allowing the effects of event history to potentially change over time (e.g. for predictor statistics that incorporate the time elapsed since a previous event, a clock event causes the calculation to update). Exogenous events also included disturbances causing birds to flush and daily observation end events. Altogether, there are 1115 event types possible. Coded this way, the observed data contain a sequence of 5168 events.

### (e) Support constraints and model scheme

Not every event type is possible at every moment; we constrain the model to impose zero hazards for impossible events [33], as follows:

(i) Only males currently present may interact or depart.
(ii) Solicitations are impossible if females are absent.
(iii) A male may depart the lek, or initiate or receive an attack, only if he is available: see the ‘Attack Cycle’ and ‘Copulation Cycle’ in figure 1. Although a pair of males in an attack cycle are unavailable, other available males may continue to act. For instance, multiple fights or copulations may be ongoing simultaneously.
(iv) At a few time points, more than one unidentified ‘X’ male is engaged in interaction. For tractability, all X males act as a single bulk-actor, which can potentially be simultaneously interacting with multiple partners, unlike other male types. X males are considered available for interaction if at least one is present.

### (f) Fixed effects for the base intercept-only relational event model

We first selected the best-performing base intercept model with fixed effects for individual-level variability in intrinsic hazards of behaviours, as well as among-category differences in hazards between identified, ‘U’ and ‘X’ males. We fitted effects for attack and receive hazards, as well as the hazards of initiating different ‘response’ events (short fights, chases and face-offs), disengagements and departures (see electronic supplementary material, table S2, for detailed descriptions of the fixed effects included in all models).

Keeping all other fixed effects in common, we tested the following two different formulations for fixed effects for solicitation hazard: (i) an ‘Aggression’ model, in which we fitted a single base solicitation hazard common to all males, forcing the model to use male–male fighting events to predict variation in solicitation; and (ii) a ‘Differential Attractiveness’ model, in which we fitted separate fixed effects for solicitation hazard for each male that was ever solicited during the season. Males that never mated were assigned the base hazard. This model allows for the possibility that individual males may possess intrinsically attractive attributes influencing their propensity to be solicited, independent of their fighting behaviour.

### (g) Model statistics

To construct the alternative models, we created a set of 301 possible predictor statistics representing potential mechanisms by which histories of fighting behaviours and other events—such as female presence, previous copulations, exogenous disturbances and covariates like distance between males—might influence the hazards of five different kinds of events: attacks, disengagements, solicitations, interruptions and departures (for a full list of statistics with descriptions, see electronic supplementary material, table S1). We developed the statistics based on observable aspects of individuals’ behaviour and information about these behaviours plausibly available to each bird. Avoiding assumptions regarding individual social memory, we incorporated statistics based on pairwise interaction history as well as those based on overall counts, and we tested these across multiple temporal scales, ranging from seconds to cumulatively across the observation period. Due to the REM’s construction, this results in numerous predictors to explore despite our relatively simple questions. For example, a particular metric like win–loss record for a focal male will be associated with several operationalized predictors. First, there are scopes of effect: general win–loss rate and pairwise win–loss rate with respect to each other male. Then, these scopes of effect may apply over multiple timescales (cumulative, yesterday and so far today). Finally, each combination applies to each relevant response variable—in this case, solicitations, interruptions, attacks and disengagements—yielding 24 win–loss record predictors to test in the full model (electronic supplementary material, table S1). Electronic supplementary material, figure S2, illustrates the categories of predictors and their relationship to the response variables.

Statistics relating to solicitation hazards consider only events that occurred in the presence of females. We do not, therefore, directly test for higher-order effects of fighting or other activity on male display not mediated by female mate choice. For example, we do not examine whether fighting that occurs with females absent might cause fatigue, negatively affecting display effort.

Statistics pertaining to copulation interruption only apply at times when a male has begun copulating. Therefore, these statistics can be thought of as testing for event-history-by-copulation interaction effects (i.e. given that a male is copulating, what is the effect of the predictor statistic on the hazard of attack, in addition to any non-context-dependent effects?). Finally, on the first and on the ninth day—which is missing a true consecutive previous day due to poor weather—we simply set statistics that count events from the previous day to their base value, usually zero.

### (h) Event history simulations

We used estimated coefficients from the final alternative REMs to simulate new event data for each day of the observation period. Beyond goodness-of-fit metrics, this approach allows us to compare how well different model formulations can approximate the actual observed grouse behaviours. For example, although the REMs give insight into what fighting behaviours, or other event histories, influence a male’s propensity to be solicited, they fit hazards of solicitations conditional only on when they are possible in a given moment. This does not consider that aggressive behaviours might have a large, emergent effect on overall mating success by causing solicitations to be impossible for substantial stretches of time.

To generate simulated data, we calculate hazards for all possible next events. Specifically, the hazard for each event is determined by exponentiating the sum of the applicable fixed effects and predictor values multiplied by their respective REM coefficients (equation (2.1)). For example, employing the best-fitting ‘Differential Attractiveness’ model (electronic supplementary material, table S3), we can calculate the hazard of the event ‘male 1 departs’. The departure fixed effect coefficient is −8.54, giving a baseline hazard of exp(−8.54) = 0.0002 s^−1^ (mean waiting time approx. 5000 s). If females were present, the predictor value for female presence would be 1. The coefficient is −1.37, so the hazard would be smaller: exp(−8.54 + 1 × −1.37) = 0.00005 s^−1^ (i.e. the male’s departure would be less likely). Event history also modifies hazards; for instance, if male 1 lost a chase 9 s ago, the predictor *gen.lose.chase.rec.depart* would apply (electronic supplementary material, tables S1 and S3; *lose chase recency*, figure 1E). Calculated as 1/sqrt(no. of seconds) since the focal male lost a chase, and with a coefficient of 2.84, this would increase male 1’s departure hazard to exp(−8.54 − 1.37 + (1/3) × 2.84) = 0.00013 s^−1^.

With the hazard of every possible next event calculated, following from the model’s assumptions, the next simulated event’s timing is chosen randomly from an exponential distribution with the mean 1/(total hazard of all possible events), and the identity of the next event is chosen as a random categorical variable weighted by its hazard’s proportion of the total hazard. For each simulated day, we fed in observed data from the previous day (‘yesterday’ being the longest relevant timescale identified in our models; see §3). We also used a scaffold of ‘scheduled’ exogenous events based on values observed on the focal day for: first arrival times for each male present, female entry and exit times and the video end. If a male departed the lek during the simulation, we used the average observed interval of absence for that male as the mean waiting time of an exponential distribution to schedule their potential return. We calculated summary counts and network metrics for simulated data using the sna package in R [61,64].

### (i) Simulation experiments

In addition to validating the REMs, we used simulation to explore how variation in individual attributes might affect lek dynamics. Using the coefficients of the best-fitting ‘Differential Attractiveness’ model (the preferred alternative formulation; see §3), we simulated data for a simplified lek consisting of six territory-holding males, excluding non-territory holders and juveniles. All males were assigned the mean attack, disengagement and solicitation hazards of territory-holding males that achieved any mating success in the observed data. Simulations assumed no early departures, the observed mean duration of female presence and the mean observation period before all birds flushed. We conducted simulations under a control condition and the following three experimental treatments: (i) increased intrinsic attack rate, (ii) increased intrinsic disengagement rate, and (iii) both combined. For each treatment, we increased the focal male’s intrinsic hazard by one s.d. above the mean while keeping the other males unchanged. These treatments were tested under the following two scenarios: an average-susceptibility focal male (mean intrinsic incoming attack hazard) and a high-susceptibility focal male (mean plus one s.d.). Each simulation run consisted of seven consecutive days of behaviour with a 1-day burn-in period. We conducted 1000 simulations per condition, aggregating metrics across all days for each simulated week. For the focal male, we calculated total fights, win rate, time available to mate, copulation interruption rate, solicitations and mating success (uninterrupted copulations). Interruption rate calculations excluded runs with three or fewer solicitations (802 of 8000); other metrics included all runs.

## 3. Results

### (a) Is fighting part of the display?

The best-fitting ‘Differential Attractiveness’ model is definitively preferred over the best-fitting ‘Aggression’ model by BIC (electronic supplementary material, tables S3 and S4; see appendix A1 for model selection protocol and analysis of model performance). In other words, the model which assumes that variation in solicitation is determined largely by male–male aggression does a substantially inferior job in explaining the observed behavioural data than the model which allows for differential intrinsic male attractiveness to explain variation in solicitation hazards. Furthermore, the final set of predictor statistics comprising the best-fitting ‘Differential Attractiveness’ model does not include any fighting-related effects on solicitation; predictor statistics like perpetrating copulation interruptions and winning fights were not necessary to explain solicitations for males to mate, regardless of the temporal scale of the event history. The number of copulations previously that day (indicative of day-level variation in display quality, or possibly mate choice copying [65]) and time of day are sufficient to explain much of the variation in solicitation hazards once variation in intrinsic attractiveness is accounted for (figure 2C and electronic supplementary material, figure S3).

These findings are corroborated by our simulations. The ‘Aggression’ model, constrained to use fighting predictors to explain variation in solicitation hazards, performs much more poorly at predicting the distribution and number of copulations per male for the observation period (figure 3).

In addition to superior performance in predicting solicitations, the ‘Differential Attractiveness’ model reveals that males are less likely to start fights, more likely to end fights sooner and less likely to depart the lek when females are present (figure 2F). This strongly suggests that male sage-grouse have evolved to reduce aggression in favour of display in the presence of females, providing direct evidence against the hypothesis that females have evolved to use male aggression to inform their mate choices.

### (b) The indirect effects of fighting on mating success

The REM indicates that fighting begets more fighting, with previously engaged males more likely to fight again. In general, being attacked (‘receiving’) and losing fights increase the risk of being attacked in the future (figure 2A). The only category of interaction that does not greatly increase a male’s propensity to be attacked is ‘attacking + winning’ (i.e. winning fights that it started; figures 2A and 4). Additionally, males who have just won a fight are more likely to start another fight (win recency ‘initiator effect’; figure 2A).

Pairs of males with more asymmetrical records of fighting outcomes are more likely to interact than more evenly matched pairs (absolute value of pairwise win–loss difference for the day; figure 2A), substantially driven by the pattern of territorial males attacking and chasing juvenile/unidentifiable males.

Besides the effects of event histories on incoming attack hazard, we also found significant individual variation in fixed effects for baseline hazard of receiving attacks (electronic supplementary material, tables S2 and S3), as well as a strong effect of pairwise centroid distance, with closer males being much more likely to interact (figure 2A).

There are several factors that influence the outcomes and durations of fights: pairwise win/loss rate today—and to a slightly lesser extent, yesterday—predicts wins (being the first to disengage) in future pairwise contests (figure 2B). Starting a fight is associated with winning (figure 2B). The remaining effects on disengagements (figure 2B) predict the duration of fights. Disturbances abruptly end fights, and female presence is associated with shorter fights overall.

The model also identifies predictors that significantly influence the hazard of copulation interruptions above the background risk of being attacked (figure 2D). The predictor statistic ‘interruption by territory holders’ indicates that copulation interruptions are most often caused by territory-holding males. It also reveals that starting a copulation greatly increases the hazard of being attacked, indicating that copulation is a strong trigger for aggression from others: the coefficient of 3.8 translates to an exp(3.8) ≈ 45-fold increase in an individual’s baseline hazard of being attacked. Furthermore, interestingly, the overall interruption hazard as well as the identity of the interrupting male is highly influenced by the copulating male’s pairwise history of getting attacked by, and losing to, those other males (‘receive + lose’ count) on the previous day (figure 2D and electronic supplementary material, figure S4).

Finally, there are several social effects on lek departure. Males are unsurprisingly more likely to remain on the lek when females are present (figure 2E,F). Recent losses in chases greatly increase departure hazard (figure 2E), with the losing male often being chased off the lek. Interestingly, ‘receive + win’ recently also predicts lek departure (figure 2E). However, because the effects of coefficients are multiplicative, these influences of fighting on the absolute hazard of departure are greater once females have left for the day.

Our results are highly suggestive that aggressively driving off competitors does not itself explain the observed variation in mating success. None of the males ever chased off the lek in our dataset mated (*n* = 14), but many other non-mating males were never chased off (*n* = 12). While fighting to drive off competitors (to establish and maintain a territory) likely contributes to securing mating opportunities, we cannot speak to that here since the lek was already formed at the start of our observations.

Though fighting does not directly influence females’ mate choice, we find it can affect mating success in the following two key ways: by limiting time available for mating (males cannot mate while fighting) and by influencing the risk of copulation interruption. Territory-holding males spent a large and considerably variable proportion of their time in front of females fighting, averaging 9.37% (±11.51) with a single-day maximum of 64.1% (electronic supplementary material, figure S5). It is likely, therefore, that a male’s tactical ability to minimize fighting and avoid conflicts that increase the risk of interruption is important for mating success. For example, on 1 April 2014 (electronic supplementary material, figure S4), male 4 spent 15.3% of his 80 min in front of females fighting, resulting in only one uninterrupted copulation out of ten attempts. In contrast, male 3, who was less aggressive and less susceptible to attack, secured three uninterrupted matings despite receiving only four solicitations.

**Figure 5.**
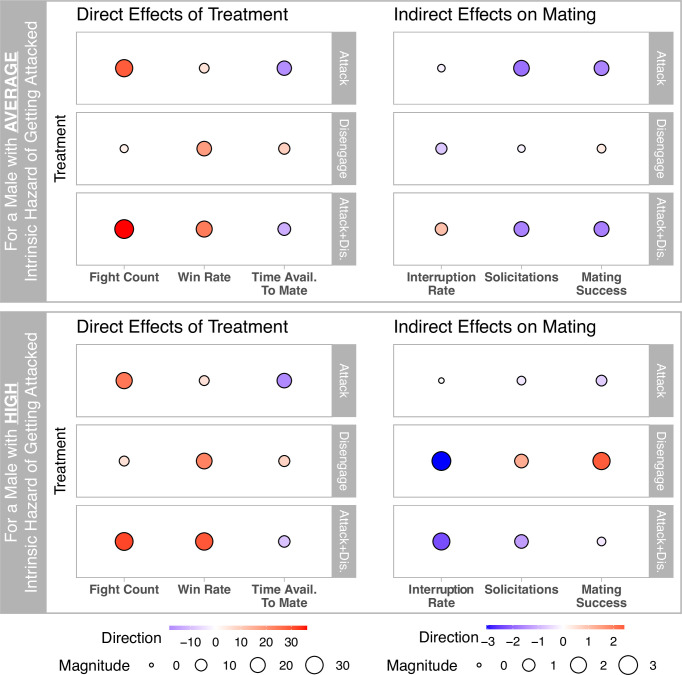
Effects of three experimental treatments (increased intrinsic attack rate, disengagement (win) rate or both) for a focal male on a simulated lek in two scenarios: average intrinsic susceptibility to be attacked (upper panels) and high susceptibility (lower panels). Bubbles show treatment effects (difference from mean control value) as *z*-scores, based on 1000 replicates per condition (electronic supplementary material, figure S13). Note scales differ between direct treatment effects (left) and downstream effects on mating (right). Simulations used coefficients from the best-fitting ‘Differential Attractiveness’ REM.

Our simulation experiment results further support the view that fighting should be avoided, or at least ended quickly and decisively. The effects of increasing intrinsic attack and disengagement rates can be parsed into the direct effects of the treatments and the downstream, indirect effects on overall mating success (figure 5). Direct treatment effects, such as increased fight frequency with higher attack rates, or improved win rates with higher disengagement rates, were evident across both average and high intrinsic susceptibility cases and were largely independent of the focal male’s susceptibility to being attacked. However, baseline metrics differed significantly between susceptibility cases: males with higher intrinsic susceptibility experienced more fights, lower win rates and less time available to mate, resulting in higher interruption rates, fewer solicitations and reduced mating success compared to males with average susceptibility (electronic supplementary material, figure S13).

Effects of treatments on mating success, mediated by time available to mate and fight histories, appear biologically relevant (figure 5, right side). For males with average susceptibility, increased attack rates reduced total mating success over the seven consecutive simulated days by 0.31 (± 0.19 SE) copulations on average due to less time available for solicitations. In contrast, increased disengagement rates positively impacted mating success, particularly for high-susceptibility males, by reducing interruption risk and increasing available mating time, leading to an extra 0.4 (± 0.16 SE) successful copulations on average. Combining increased attack and disengagement rates generally reduced mating success, especially for average-susceptibility males (−0.32 ± 0.19 SE copulations), as the benefits of higher disengagement (i.e. having a better winning record) were overwhelmed by the negative impact of more frequent fights.

## 4. Discussion

Our analysis reveals that fighting behaviour does not directly predict copulation events or overall mating success in a greater sage-grouse lek. In the best-fitting ‘Differential Attractiveness’ model, we recovered no evidence that fighting is itself attractive, directly informative or functions in concert with display to contribute to the solicitation of copulation by females. In fact, males are less likely to start fights and more likely to end them sooner in the presence of females on the lek (figure 2F). Rather, matings more likely depended on the unexplained variation among individual males’ propensity to be solicited. The importance of intrinsic male variation is consistent with extensive evidence that elements of the famously elaborate display in greater sage-grouse, and those of other grouse species, are associated with mating success [57,66,67]. However, our result that fighting is not attractive may seem counter-intuitive, since fighting behaviour in these systems is often also correlated with mating success in aggregate [15,16,18,54]. Indeed, we recover a similar pattern in our aggregate data (electronic supplementary material, figure S6). However, the direct causal, mechanistic relationship potentially implied by this correlation does not hold when we account for the sequence and timing of events in the REM.

The main influences of fighting on mating success are indirect, affecting the time available to display and to mate and the risk of copulation interruption (figures 3 and 5 and electronic supplementary material, figure S4). The best strategy for males in terms of maximizing mating opportunities seems to be to not start fights unless they are going to win. The REM and subsequent simulation analysis uncovered an underappreciated, yet significant risk of initiating fights: fights can consume a lot of time and lead to further fights, resulting in emergent, self-sustaining webs of conflict that are difficult to diffuse [63,68] (figures 4 and 5). This showcases the utility of the simulation experiments: based on the hazard calculations alone (figure 4), it may seem that ‘attacking + winning’ has no consequences, when in fact this can still lead to significant wasted time and missed mating opportunities (figure 5). It is not clear that initiating fights would benefit an attractive male, but our results suggest the possibility that engaging other males’ time through fighting is a way for less attractive males to potentially increase their own relative probability of mating, thus flattening the skew in mating success. Given that individual males cannot totally control whether they end up fighting, winning contests and having a history of winning is also important for deterring interruptions, since losing fights contributes greatly to interruption risk (figures 2D and 4).

The REM also indicated significant variation in individual males’ intrinsic hazard of being attacked (electronic supplementary material, tables S2 and S3), and our simulations revealed this to have serious implications for their propensity to become enmeshed in cycles of aggression and for their vulnerability to interrupted copulations (figure 5 and electronic supplementary material, figure S13). This variation in attack susceptibility could be associated with a covariate that other males can evaluate without needing to interact (such as body size or display behaviour), or which other males could be aware of due to history beyond the scope of our study, such as a competitor’s age or the outcome of pre-season (or possibly off-lek) fighting. Alternatively, or additionally, variation in individual-level hazard of being attacked could be a proxy for some aspect of territory defence. Although metrics such as a territory’s position relative to the centre of the lek or a territory’s relative size have previously been associated with mating success in other lekking grouse [16,54], the territories in our study were highly clustered (more-or-less equally central) and highly stable throughout the observation period (electronic supplementary material, figure S14), making it difficult to disentangle males’ identity and territory effects. However, our results suggest that females are not attracted to territory defence ability *per se*, but rather to the displays that successful territory defence facilitates, as some have speculated [50,69]. Given the strong effect of pairwise centroid distance on the risk of getting attacked (figure 2A), behaviour such as attacking and winning may represent a constant underlying requirement for getting the space necessary for display and successful copulation. This is possibly why we do not observe a direct effect of these fighting behaviours on solicitation, but why we recover an association between general aggression and mating success in aggregate (electronic supplementary material, figure S6).

Overall, male aggression has a complex relationship with female mate choice. Fighting is not attractive. Fighting and copulation interruption are forms of sexual coercion [11], distracting attractive males away from displaying, preventing females from evaluating those displays and, in some cases, physically preventing females from fulfilling their mate choices. However, fighting can be aligned with female choice when winning fights contributes to social stability and protects females’ ability to choose based on display through deterring interruptions and possibly maintaining territory boundaries. In short, aggression is an essential part of successful male sage-grouse reproductive behaviour, but to be used successfully, it must be deployed in a context-sensitive manner to reduce the risks of disruption to display and copulation. Given our finding that fighting is unlikely to be an aspect of display, our analysis strongly favours the idea that the evolved intrasexual male behavioural mechanisms necessary for regulating aggression and promoting female choice plays a crucial, yet underappreciated role in how leks evolve as arenas for females to make mating decisions [13,70]. The evolution of ‘social skill’ [71] and the ability to tactically allocate time and resources is clearly useful for individual male reproductive success in this system [56,57]. However, recognizing that fighting is an emergent property of the social group and not entirely within the control of any one individual, our analysis is also consistent with the hypothesis that female choice for the regulation of aggression allows a concentrated lek to stably evolve and function as a communal display ground [70], raising intriguing questions. If aggression interferes with display and often directly with mating decisions, should females avoid lek groups that display poor social regulation? Does fighting decrease lek-level (or possibly even population-level [72]) reproductive success? There is some empirical evidence from other species that disturbance to the established social structure of leks increases fighting and may decrease the overall mating success of lek members [73,74].

Our results demonstrate the REM framework’s broad applicability to difficult behavioural questions in which the order and timing of events really matter for our ultimate interpretation of underlying processes. In this case, a more traditional correlational study is inadequate to differentiate the mechanistic relationships among fighting, display and copulation events, yielding misleading results. Moreover, we can use the REM to elucidate mechanisms including indirect pathways to mating success and emergent social outcomes such as self-fuelling cycles of violence that are indeed the proximate cause of much of the aggression on the lek.

## Data Availability

Data and code are available as part of the electronic supplementary material and accessible on Dryad [62]. Supplementary material is available online [75].
